# Clinical utility of geriatric assessment tools in older patients with gastrointestinal cancer

**DOI:** 10.3389/fonc.2023.1110236

**Published:** 2023-05-31

**Authors:** Ayako Doi, Takuro Mizukami, Hiroyuki Takeda, Kumiko Umemoto, Hiroyuki Arai, Yoshiki Horie, Naoki Izawa, Takashi Ogura, Yu Sunakawa

**Affiliations:** ^1^ St. Marianna University School of Medicine, Department of Clinical Oncology, Kawasaki, Japan; ^2^ Nippon Telegraph and Telephone Corporation (NTT) Medical Center Tokyo, Department of Medical Oncology, Tokyo, Japan; ^3^ Kawasaki Municipal Tama Hospital, Department of Clinical Oncology, Kawasaki, Japan

**Keywords:** geriatric 8 (G8), instrumental activities of daily living (IADL), elderly, gastrointestinal cancer (GI cancer), chemotherapy

## Abstract

**Background:**

Geriatric 8 (G8) and instrumental activities of daily living (IADL) are recommended to predict overall survival (OS) or risk of serious adverse events (SAEs) in older cancer patients. However, the clinical utility is relatively unknown in older patients suffering malnutrition with gastrointestinal (GI) cancer, including gastric cancer (GC) and pancreatic cancer (PC).

**Materials and methods:**

We retrospectively included patients aged ≥65 years with GC, PC, and colorectal cancer (CRC) who received a G8 questionnaire at first visit from April 2018 to March 2020. The associations between G8/IADL and safety or OS were assessed in patients with advanced/unresectable tumors.

**Results:**

Of 207 patients (median age: 75 years), the median G8 score was 10.5 and normal G8 score rate was 6.8%. Both the median G8 score and normal G8 (>14) score rate numerically increased in the order of GC < PC < CRC. There was no clear association between the G8 standard cutoff value of 14 and SAEs or OS. However, OS was significantly longer in patients with G8 >11 than in those with G8 ≤11 (19.3 vs. 10.5 months, *p* = 0.0017). Furthermore, OS was significantly better in patients with normal IADL than in those with abnormal IADL (17.6 vs. 11.4 months, *p* = 0.049).

**Conclusion:**

The G8 cutoff value of 14 would not be clinically useful in patients with GI cancer for predicting OS or SAEs; however, the cutoff value of 11 and IADL may be useful to predict OS for older patients with GI cancers including GC and PC.

## Introduction

The number of older cancer patients has increased worldwide as the population has aged and older patients aged ≥65 years account for more than 70% of all cancers in Japan, which represents elderly society (1). Serious adverse events (SAEs) from chemotherapy (CT) occur more frequently in older patients because they often have several comorbidities as well as compromised physical and organ function (2). Therefore, it is important to consider the tolerability of CT in older patients. The International Society of Geriatric Oncology (SIOG) and the American Society of Clinical Oncology (ASCO) have recommended a geriatric assessment (GA) scale for older cancer patients to predict overall survival (OS) and treatment-related toxicity in consideration of optimal disease management (3–5). Moreover, both societies emphasize the importance of implementing GA-based interventions. However, it may be difficult to perform a complete GA routinely in all patients as it requires some effort and expense in a busy clinical practice.

There are several screening tools for predicting functional impairment and selecting patients for GA. One of these tools is the geriatric 8 (G8), which mainly consists of items from the mini-nutritional assessment (MNA) questionnaire (6). The G8 has been validated with respect to predicting survival in various cancers (7, 8). A cutoff score of ≤14 is considered abnormal and is associated with poor prognosis (6, 8). The instrumental activities of daily living (IADL) is also recommended as a part of the GA (9). The IADL is directly linked to independence of daily living and can affect the feasibility of CT. The ASCO guideline for geriatric oncology has recommended a G8 assessment for older cancer patients receiving CT as practical for the management of toxicity. In addition, IADL was also proposed to assess of functionality at a minimum setting of GA for those patients (5).

There have been several reports about the utility of G8 in older patients with solid tumors, including gastrointestinal (GI) cancers, showing that 8.2%–89% exhibit an abnormal G8 score (6, 8, 10–20). However, most reports about GI cancer related to colorectal cancer (CRC) and there has been little evidence regarding the relationship between G8 and OS or SAE in older patients with gastric cancer (GC) or pancreatic cancer (PC) (6, 8, 14–18). Previous studies have reported that G8 score and its accuracy vary significantly according to cancer type. In addition, it has been shown that a G8 cutoff value of 10.5–12 might predict prognosis in older patients with various cancers (11, 13, 19, 20). However, it is questioned whether the G8 cutoff value should be the same for all cancers; in particular, it is expected that the cutoff value should differ for GI cancers, as patients often suffer from malnutrition.

To address this question, we examined the association between G8/IADL and clinical outcomes to evaluate the clinical utility of GA tools in older patients with GI cancer, including GC and PC.

## Materials and methods

### Patients

We included patients aged ≥65 years with GC, PC, and CRC who visited for treatment and received a G8 questionnaire at first visit from April 2018 to March 2020 in Department of Medical Oncology, St. Marianna University Hospital in Japan. The G8 questionnaire is prospectively performed in patients aged ≥65 years at baseline in clinical practice at our hospital.

This study was approved by the institutional review board of St. Marianna University School of Medicine bioethics committee (No. 5465), and the need for informed consent was waived as it was a retrospective study with data analyzed anonymously. All procedures were performed in accordance with relevant guidelines and regulations.

### G8 assessment and other measures

We calculated G8 score from the G8 questionnaire form for each registered patient. As the G8 score of ≤14 was considered abnormal according to the conventional classification, normal G8 was defined as a G8 score of >14 (6). We also collected IADL data using the Lawton IADL scale that assesses the ability to use telephone, shopping, food preparation, housekeeping, laundry, mode of transportation, responsibility for own medications, and the ability to handle finances. Women were scored on all 8 areas, while 3 areas (food preparation, housekeeping, and laundry) were excluded for men (9). Normal IADL was defined as full score (8 score for women and 5 score for men) and score other than full was considered abnormal.

We retrospectively collected data regarding patient characteristics, dose reduction, treatment discontinuation, SAE, and OS as clinical outcomes from medical records. We evaluated CT induction rate and these clinical outcomes for adjuvant and palliative CT, respectively. The AEs were assessed according to the National Cancer Institute Common Terminology Criteria for Adverse Events (NCI-CTCAE version 4.0). SAEs were defined as grade 3–5 hematologic and non-hematologic AEs or AEs requiring hospitalization. OS was defined as the period from the date of first visit to the Department of Medical Oncology to the date of death from any cause in patients with unresectable tumors.

### Statistical analysis

Differences in G8 score and each G8 item between patients with or without CT and between patients with GC, PC, and CRC were analyzed by the Wilcoxon test and Fisher’s exact test. In addition, differences in other measures of GA between patients with or without CT were analyzed by Fisher’s exact test. Differences in clinical outcomes for patients with each type of cancer were analyzed using Fisher’s exact test. Differences in clinical outcomes between normal and abnormal IADL groups, or among groups using a combination of G8 score and IADL, were also explored using Fisher’s exact test. We used logistic regression analysis to evaluate the odds ratios of clinical outcomes. OS was estimated using the Kaplan-Meier method and it was compared between two groups by a log-rank test in patients with unresectable tumors. The optimal G8 cutoff was determined using receiver-operating characteristic (ROC) curve and Youden index analysis. For all analyses, *p* < 0.05 was considered statistically significant. All data were analyzed using JMP 12 software (SAS Institute Inc., Cary, NC, USA).

## Results

### Patient characteristics and clinical outcomes

A total of 207 patients aged ≥65 years with GI cancers were included in this study. The patient characteristics are listed in **Table 1**. The median age of the patients was 75 years-old (range, 65–92) and the median BMI was 20.8 (range, 13.5–31.6). The types of cancers were CRC (52.2%), PC (29.0%) and GC (18.8%) and 115 (56.0%) patients of them were advanced stage cancers with unresectable tumors. In all registered patients, 143 (69.0%) received CT (63 with adjuvant CT and 80 with palliative CT). CT introduction rates were similar in both adjuvant and palliative setting (68.5% vs. 69.6%). In palliative setting, nine patients received molecular targeted therapy and seven of nine patients received combination therapy of cytotoxic and molecular targeted agents. Three patients received immune checkpoint inhibitor monotherapy. In patients not receiving CT, the main reasons for avoiding CT were patient decision (65.6%), poor PS (25.0%), and some overlapping reasons. Patients who were not treated with CT were significantly older and had worse PS compared with those who underwent CT (*p* < 0.0001 and *p* = 0.0002, respectively).

**Table 1:** Patient characteristics.

		All patients (n = 207), n (%)	With CT (n = 143), n (%)	Without CT (n = 64), n (%)
Age, year	Median (range)	75 (65–92)	73 (65–86)	80 (66–92)
Sex	Male	109 (52.7)	76 (53.1)	33 (51.6)
	Female	98 (47.3)	67 (46.9)	31 (48.4)
PS	0	98 (47.3)	83 (58.0)	15 (23.4)
	1	88 (42.5)	58 (40.6)	30 (46.9)
	≥2	15 (7.2)	1 (0.7)	14 (21.9)
	Unknown	6 (2.9)	1 (0.7)	5 (7.8)
BMI	Median (range)	20.8 (13.5–31.6)	21.0 (13.5–31.6)	20.0 (14.8–27.5)
Cancer type	Colon/Rectum	108 (52.2)	75 (52.4)	33 (51.6)
	Pancreas	60 (29.0)	41 (28.7)	19 (29.7)
	Stomach	39 (18.8)	27 (18.9)	12 (18.7)
Staging	Resectable	92 (44.4)	63 (44.1)	29 (45.3)
	Unresectable	115 (55.6)	80 (55.9)	35 (54.7)
Treatment	Palliative CT	80 (38.6)	80 (55.9)	–
	Adjuvant CT	63 (30.4)	63 (44.1)	–
	No CT	64 (31.0)	–	64 (100)

-, not applicable; PS, performance status; BMI, body mass index; CT, chemotherapy.

Of 143 patients with CT, the rate of those who had dose reduction at start and during CT were 38.5% and 76.2%, respectively. The rate of SAEs was 51.7%, hematological and non-hematological SAEs were both 25.9%, and discontinuation rate of CT was 17.5% (**Table 2**). The rate of SAE was significant higher in patients with palliative CT than those with adjuvant CT (80% vs. 63%, *p*=0.012), however, the rates of other clinical outcomes were similar among them. Neutropenia was the most common SAE (41.2%), while febrile neutropenia occurred in only a few patients (2.8%). Only one patient died from treatment-related complication and one patient died from overlapped other cancer.

**Table 2:** Clinical outcomes according to G8 score in patients treated with chemotherapy.

	All patients (n = 143), n (%)	With G8 >14 (n = 14), n (%)	With G8 ≤14 (n = 129), n (%)	OR (95%CI)
DR at start	55 (38.5)	3 (21.4)	51 (39.5)	2.44 (0.65–9.19)
DR during CT	109 (76.2)	12 (85.7)	96 (74.4)	0.49 (0.1–2.3)
SAEs	74 (51.7)	6 (42.9)	68 (52.7)	1.51 (0.5–4.59)
Hematological SAEs	37 (25.9)	4 (28.6)	33 (25.6)	0.86 (0.25–2.93)
Non-hematological SAEs	37 (25.9)	3 (21.4)	34 (26.4)	1.31 (0.35–4.99)
Discontinuation	25 (17.5)	0 (0.0)	25 (19.4)	–

-, not applicable; G8, geriatric 8; CT, chemotherapy; DR, dose reduction; SAEs, serious adverse events; OR, odds ratio; CI, confidence interval.

### G8 score of older patients with GI cancer

The median G8 score was 10.5 (range, 2–16) and 6.8% of patients had normal G8 score (>14; **Table 3**). According to cancer type, both the median G8 score and the rate of G8 score of >14 numerically increased in the order of GC < PC < CRC: median score, 9.5, 10.5, and 11; rate, 2.6%, 5.0%, and 10.2%, respectively (**Supplemental Table 1**).

**Table 3:** G8 score and other measures of GA.

		All patients (n = 207), n(%)	With CT (n= 143), n(%)	Without CT (n = 64), n(%)	*P*-value*
G8 score	Median (range)	10.5 (2–16)	11.5 (6–16)	10 (2–16)	<0.0001
	Normal	15 (6.8)	14 (9.8)	1 (1.6)	0.041
	Abnormal	192 (93.2)	129 (90.2)	63 (98.4)	
IADL	Normal	107 (51.7)	92 (64.3)	15 (23.4)	<0.0001
	Abnormal	81 (39.1)	37 (25.9)	44 (68.8)	
	Unknown	19 (9.2)	14 (9.8)	5 (7.8)	
Living situation	With others together	163 (78.7)	111 (77.6)	52 (81.3)	0.068
	Alone at home	42 (20.3)	32 (22.4)	10 (15.6)	
	Nursing home	2 (1.0)	0 (0.0)	2 (3.1)	

G8, geriatric 8; GA, geriatric assessment; IADL, instrumental activities of daily living; CT, chemotherapy.

*Difference in G8 score between patients with or without chemotherapy was analyzed by the Wilcoxon test. Differences in IADL or living situation between patients with and without chemotherapy were determined by Fisher’s exact test.

Patients with CT had significantly higher median G8 score than those without CT (median G8 11.5 vs. 10.0, *p* < 0.0001), however 90.2% of them treated with CT had abnormal G8 score ≤14. As background for G8 ≤14, more than half of the patients had low score with respect to food intake, weight loss, BMI, prescription drug, and self-perception of health status. Especially, the rate of zero score of weight loss and BMI item were 46.4% and 29.5%, whereas the rate of perfect score of them were only 24.6% and 24.2%, respectively (data not shown). At the same time, patients with CT had significantly better mobility (*p* < 0.0001), less neuropsychological problems (*p* = 0.0005), and were younger (*p* < 0.0001) compared to those without CT.

### Association between G8 score and clinical outcomes

We assessed the association between G8 score and dose reduction or toxicity in 143 patients with CT. There was no significant difference in dose reduction and SAEs between patients with normal and abnormal G8 scores (**Table 2**). There was also no association between the G8 cutoff and clinical outcomes for each treatment setting. Furthermore, logistic regression analysis using explanatory variables such as age, PS, and BMI showed that age and PS had significant differences in upfront dose reduction (age; *p* = 0.0003, PS; *p* = 0.048) and patients with GC and PC had higher rate of upfront dose reduction than those with CRC (Upfront dose reduction: GC 59.3%, PC 41.5%, CRC 28%). However, SAE or discontinuation of CT did not relate to these variables (data not shown) and the rate of SAE did not differ clearly among cancer types (SAE: GC 48.2%, PC 56.1%, CRC 50.7%).

**Figure 1:**
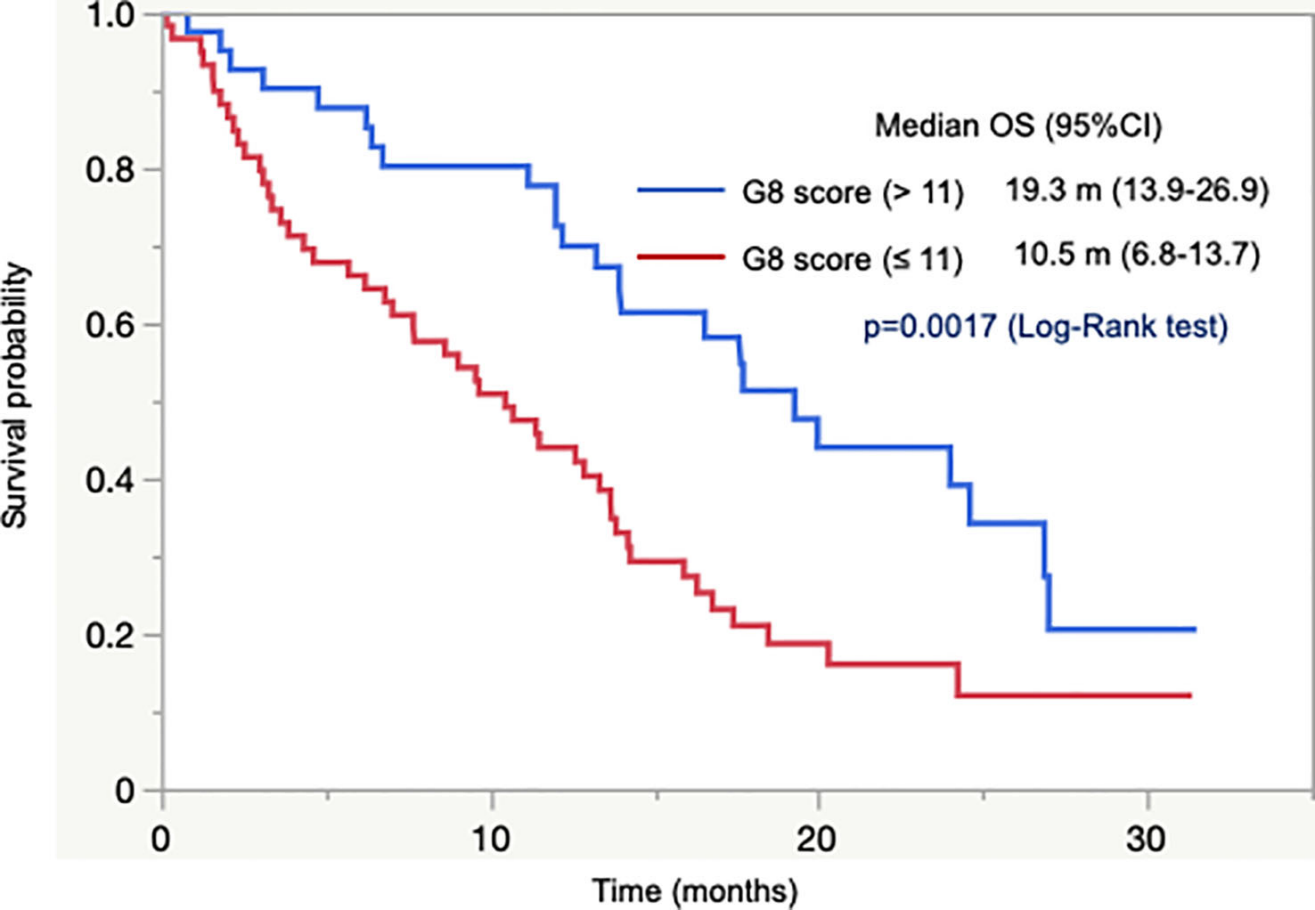
Overall survival according to G8 score of 11.

Next, we analyzed the association between G8 score and OS in 101 patients with unresectable tumors who were not previously treated with CT. OS did not differ between patients with G8 >14 and G8 ≤14. It indicated that G8 cutoff value of 14 did not predict survival. Subsequently, we conducted ROC curve analysis, which yielded a cutoff value of 11 and an area under the curve value of 0.65. According to the cutoff value of 11, OS was significantly longer in patients with G8 >11 than in those with G8 ≤11 (median OS = 19.3 months vs. 10.5 months, *p* = 0.0017; **Figure 1**). In addition, OS was significantly longer in patients with CT than in those without CT (median OS = 15.9 months vs. 6.2 months, *p* = 0.0002) as well as in patients who had abnormal G8 score with CT than in those without CT (median OS = 14.2 months vs. 6.2 months, *p* = 0.0009). In adjuvant setting, OS could not be assessed due to very few events.

These results indicated that there was no clear association between G8 cutoff value of 14 and dose reduction, safety, or survival. However, a cutoff value of 11 could predict survival in older patients with GI cancer.

### Association between IADL and clinical outcomes

We also investigated the clinical utility of IADL in this study. In all registered patients, the rate of normal IADL was 51.7%. According to cancer type, the rate of normal IADL numerically increased in the order of GC < PC < CRC: 38.5%, 53.3%, and 55.6%, respectively (**Supplemental Table 1**). The rate of normal IADL was significantly higher in patients with CT than in those without CT (64.3% vs. 23.4%, *p* < 0.0001) as well as in patients who had abnormal G8 score with CT than in those without CT (62.0% vs. 22.2%, *p* < 0.0001). The discontinuation rate of CT was higher in patients with abnormal IADL than in those with normal IADL (24.3% vs. 14.1%, OR = 1.95); however, there was no significant association between IADL and SAE and other clinical outcomes.

**Figure 2:**
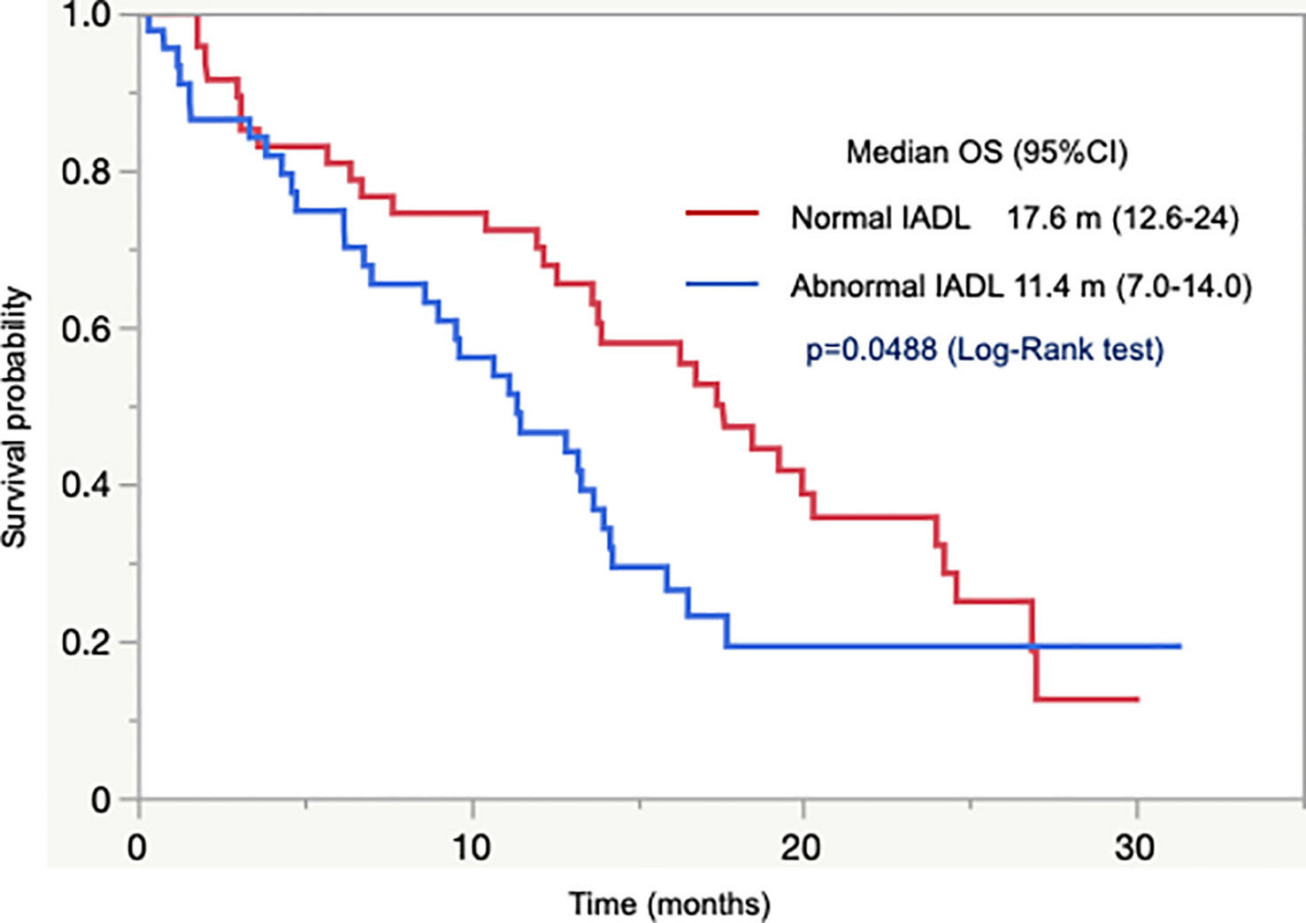
Overall survival according to IADL.

In 92 patients with unresectable tumors who were assessable using IADL, there was a significant difference in OS between patients with normal and abnormal IADL. Patients with normal IADL had longer OS than those with abnormal IADL (median OS 17.6 months vs. 11.4 months, *p* = 0.049; **Figure 2**). In addition, the OS was numerically longer in patients with normal IADL than in those with abnormal IADL in 86 patients with abnormal G8 (median OS 16.8 months vs. 11.4 months, *p* = 0.13).

### Clinical outcomes according to group in combination with G8 score and IADL

**Figure 3:**
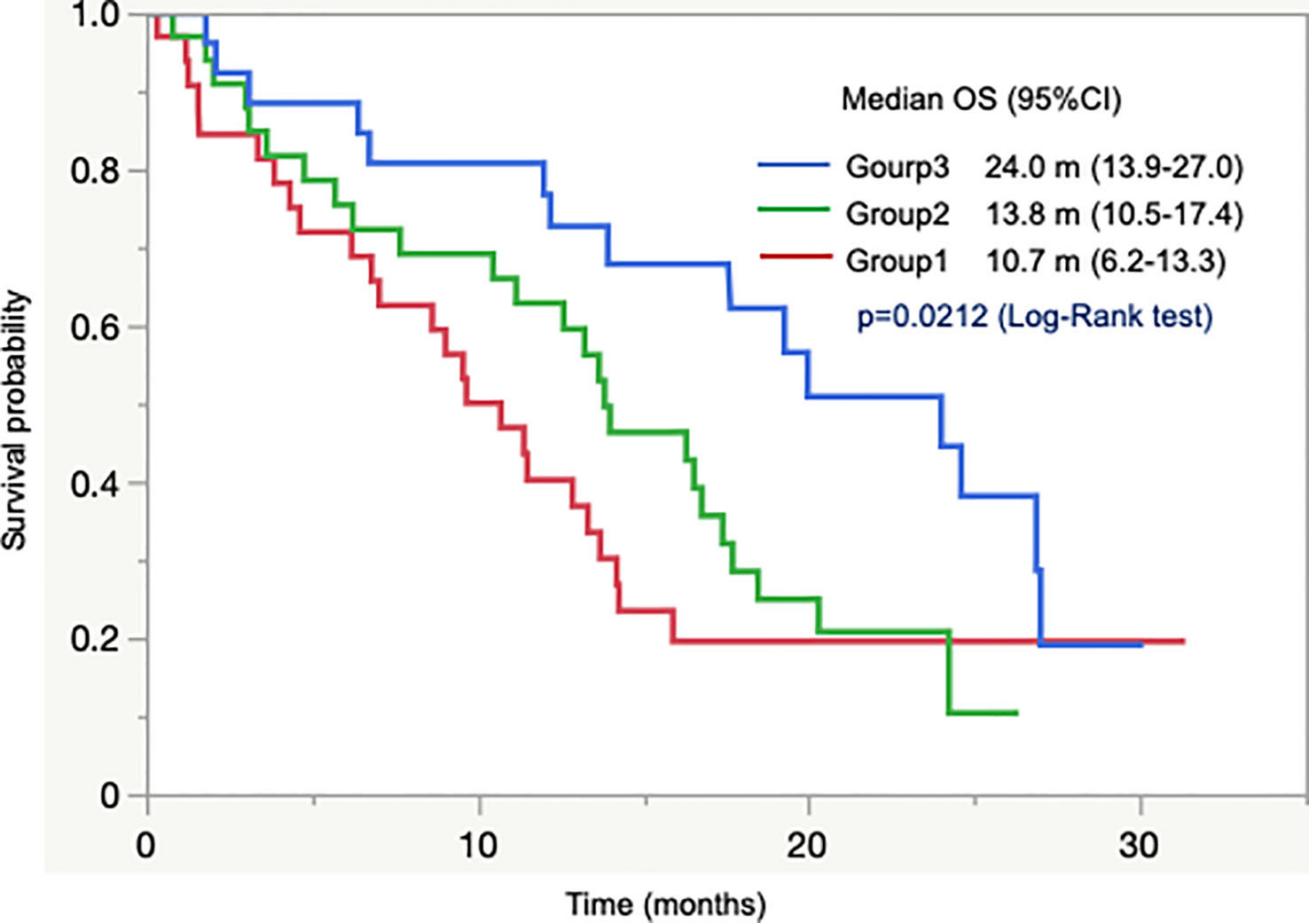
Overall survival according to combination of GB and IADL group.

We performed an exploratory assessment of clinical outcomes according to the group in combination with G8 score and IADL. We classified patients into three groups based on a combination of G8 score, wherein we defined 11 as its cutoff, and IADL: group 1, abnormal for both G8 score and IADL (n = 59); group 2, abnormal for either G8 score or IADL (n = 76); and group 3, normal for both G8 score and IADL (n = 53). Among the 188 patients assessed with both G8 score and IADL, group 1 had significantly lower rate of CT introduction than other groups (42.4% in group 1, 77.6% in group 2, and 84.9% in group 3, *p* < 0.0001), whereas there was no significant difference in both SAEs and upfront dose reduction rate among the groups. However, the discontinuation rate of CT was numerically the highest in group 1, followed by groups 2 and 3 (28.0%, 15.3%, and 13.3%, *p* = 0.27). Among 92 patients with unresectable tumors, OS differed significantly among groups; patients in group 3 had the longest OS, followed by groups 2 and 1 (median OS 24.0 months, 13.8 months, and 10.7 months, respectively, *p* = 0.021; **Figure 3**).

## Discussion

Our study showed that the percentage of normal G8 score >14 was only 6.8% in older patients with GI cancer. The rate was much lower in patients registered in our study compared with median rate of 18.2% (8.2%–31.6%) in previous reports involving older patients with most solid tumors (6, 8, 10–13, 17–20). In addition, G8 cutoff value of 14 did not predict either OS or SAEs, which is inconsistent with previous reports (6, 8). Furthermore, patients who received CT with relative safety had a better prognosis compared to those without CT, even in patients with G8 ≤14. These results indicate that conventional cutoff value of 14 may not be clinically useful in older patients with GI cancer, most of whom have G8 ≤14 due to severe malnutrition.

There are several reasons of much lower normal G8 score rate of this study. One reason for low G8 score in patients with GI cancer is malnutrition and low BMI due to digestive symptoms, such as nausea and appetite loss. G8 score consists of an MNA questionnaire that primarily focuses on nutrition; therefore, nutritional status is well reflected by the score. In this study, the score for items regarding digestive symptoms was lower compared to patients with various cancer types including non-GI cancer, whereas the score for other items was similar (13). A previous study showed that the proportion of abnormal G8 score was significantly different among cancer types (11). In particular, patients with gastroesophageal cancer had the highest frequency of malnutrition compared to other cancer types. Patients with GC suffered from malnutrition twice as often as those with CRC, indicating that nutritional status differs according to location among GI cancers (21). In this study, patients with GC accounted for 18% and they had the lowest median G8 score (9.5) and the lowest rate of normal G8 score (2.6%) followed by those with PC and CRC. In a previous study, the rate of G8 >14 was even lower than 8.2% for most patients with GC (17). Thus, older patients with GI cancer, especially GC, have a greater risk of malnutrition leading to lower G8 score. Second, the mean BMI values in older Japanese people and patients in this study are about 22 and 20.8 which are lower than those in Western people (22, 23) and the rate of zero score of BMI item of G8 is 29.5%, whereas the rate of perfect score of BMI is only 24.2% in this study, that is also the reason to decrease the rate of normal G8 score. BMI is known to vary among ethnic groups and there is considerably less information about G8 scores in older Asian patients with GI cancer. Accordingly, it is well worth considering the cutoff values in such individuals because there are only a few reports of small study. As most of them were reported from Japan (13, 19, 20), we think the G8 cutoff score of 14 might not be appropriate for not only older patients with GI cancers but also Asian population who have lower BMI. In those cases, it is recommended to use GA tools that are unaffected by nutrition status in addition to G8 score. Finally, some reports included patients treated with chemotherapy in clinical study, who had relatively better condition compared to patients in clinical practice. On the other hand, our study was based on real-world data including worse conditioned older patients with or without chemotherapy.

The IADL is an important GA tool that is directly linked to independence of daily living. The SIOG and ASCO guidelines recommend both IADL and G8 for older patients with cancer receiving CT because IADL consists of question regarding the ability to care for oneself, including responsibility for taking medications, which can affect the feasibility of CT. Some reports have indicated an association between IADL and SAEs or OS; however, reports regarding clinical utility of IADL in older patients with GI cancer is more limited than that of G8 (24–26). In our study, the rate of normal IADL was significantly higher in patients with CT compared to those without CT regardless of G8 score. Moreover, OS varied significantly with IADL and OS was numerically longer in patients with normal IADL compared to those with abnormal IADL even cases with abnormal G8. In our exploratory analysis using combination of G8 cutoff value of 11 and IADL, patients with both normal G8 and IADL had significantly longer OS compared to other patients. On the other hand, patients with G8 ≤ 11 and abnormal IADL had higher rate of CT discontinuation and worse prognosis than other patients, however, there was no difference in both SAE and upfront dose reduction among them. Some patients with G8 ≤ 11 discontinued CT due to PS decline, unexpected complication and/or persistent moderate adverse effect less than grade 3. As a result, we recommend further upfront dose reduction and early judgment of second dose reduction for them. We might treat these patients more carefully than others.

Older patients with GI cancer—especially GC and PC—frequently suffer from malnutrition which tend to be overrated in G8 frailty, therefore one report evaluated the utility of modified G8 (27). However, there was no difference in SAE among cancer types. Perhaps due to worse condition from malnutrition, patients with GC and PC had higher rate of upfront dose reduction than those with CRC and the dose reduction might prevent SAE. In contrast to G8 score, IADL is scarcely affected by malnutrition and may be more clinically useful for predicting prognosis in such individuals. Overall, these findings suggest that GA tools less affected by malnutrition are of potential clinical utility to determine the optimal treatment plan more accurately in older patients with GI cancer. In addition, combined scoring using optimal G8 cutoff and IADL may be more useful because each tool addresses a limitation of the other. Further prospective research is needed to evaluate the utility of combination scoring.

On the basis of these results, this study is meaningful for a warning that there may be a difference in G8 cutoff value between GI and non-GI cancers and/or among ethnic groups due to the difference of nutritional status and/or body type related to BMI. Our study focused on GC, PC and CRC with each cancer data although previous similar reports have more miscellaneous cancer patients including non-GI cancers. There is less information of GA about older GI cancer patients, especially GC patients, therefore our results would become significant reports.

There are several limitations in this study. First, this was a retrospective study with GC, PC, and CRC patients in a single institution. Therefore, there were several biases including patient selection and the various treatment regimens that could affect OS and SAE frequency and OS analysis was performed in patients with three GI cancer types together. However, previous many studies regarding the G8 scoring for older patients indicated that G8 cutoff score of 14 is useful to predict prognosis in population composed of various cancers including both GI and non-GI cancers with any treatment; therefore, we think that we don’t have to arrange same patients’ background strictly. On the other hand, it is important to evaluate the utility of G8 for each GI cancer, therefore, we would like to conduct a larger cohort study in future. Second, treatment choice was affected by multiple factors regardless of the G8 score or IADL in clinical practice; therefore, it would be necessary to verify the efficacy of G8 scoring or IADL for judging the feasibility of CT by randomized controlled study. Third, as the sample size of the patients with G8 >14 was small, we could not compare OS between patients with G8 >14 and G8 ≤14. Therefore, we used Pearson’s correlation analysis to evaluate the association between G8 and survival time. Finally, we could not obtain detailed information about intervention such as nutritional guidance and rehabilitation. However, we obtained data regarding dose reduction, which was one of the interventions for patients with G8 ≤14.

## Conclusion

This study demonstrated that there was no clear association between G8 cutoff value of 14 and SAEs or OS in older patients with GI cancer. The conventional G8 cutoff score would not be clinically useful in those patients due to severe malnutrition. On the other hand, the cutoff value of 11 and IADL, which is rarely affected by malnutrition, may be useful tools to predict OS in clinical practice for older patients with GI cancers including GC and PC. Owing to some limitations of this study, further large prospective studies are needed for better decision-making in caring for older patients with GI cancer.

## Data availability statement

The original contributions presented in the study are included in the article/**Supplementary Material**. Further inquiries can be directed to the corresponding author.

## Ethics statement

The studies involving human participants were reviewed and approved by St. Marianna University School of Medicine bioethics committee. Written informed consent for participation was not required for this study in accordance with the national legislation and the institutional requirements.

## Author contributions

AD and YS contributed to the study conception and design. AD collected the clinical data. Data were interrupted by AD and TM. AD and YS wrote the manuscript. All authors contributed to the article and approved the submitted version.
