# Supplementary material for: Clinical utility of geriatric assessment tools in older patients with gastrointestinal cancer
Source: Front Oncol. 2023 May 31;13:1110236. doi: 10.3389/fonc.2023.1110236 (PMC10264801; doi:10.3389/fonc.2023.1110236)
Supplement: Supplementary file 1 [file Table_1.docx]

Supplementary Material

The clinical utility of geriatric assessment tools in older patients with gastrointestinal cancer

Ayako Doi ^1^, Takuro Mizukami ^1,2^, Hiroyuki Takeda ^1^, Kumiko Umemoto ^1^, Hiroyuki Arai ^1^, Yoshiki Horie ^1^, Naoki Izawa ^1^, Takashi Ogura ^1,3^, Yu Sunakawa ^1*^

*** Correspondence:** Yu Sunakawa: y.sunakawa@marianna-u.ac.jp

# Supplementary Table 1. G8 score and other measures of GA by type of cancer

|  |  | CRC (n = 108), n (%) | PC (n = 60), n (%) | GC (n = 39), n (%) |
| --- | --- | --- | --- | --- |
| G8 score | Median (range) | 11 (4–16) | 10.5 (2–16) | 9.5 (6–15) |
|  | Normal | 11 (10.2) | 3 (5.0) | 1 (2.6) |
|  | Abnormal | 97 (89.8) | 57 (95.0) | 38 (97.4) |
| IADL | Normal | 60 (55.6) | 32 (53.3) | 15 (38.5) |
|  | Abnormal | 44 (40.7) | 23 (38.3) | 14 (35.9) |
|  | Unknown | 4 (3.7) | 5 (8.3) | 10 (25.6) |
| Living situation | With others together | 82 (75.9) | 50 (83.3) | 31 (79.5) |
|  | Alone at home | 24 (22.2) | 10 (16.7) | 8 (20.5) |
|  | Nursing home | 2 (1.9) | 0 (0.0) | 0 (0.0) |

Abbreviations: G8, geriatric 8; GA, geriatric assessment; CRC, colorectal cancer; PC, pancreatic cancer; GC, gastric cancer; IADL, instrumental activities of daily living.

*Difference in G8 score between patients with or without CT was analyzed by the Wilcoxon test. Differences in IADL and living situation between patients with or without CT were determined by Fisher’s exact test
